# The Press, the Working Classes, and Parliament

**Published:** 1912-11-23

**Authors:** 


### THE PRESS, THE WORKING CLASSES, AND PARLIAMENT.

WE have given of our best during a long and busy life to the cause of the hospitals, and of the sick and suffering in these islands and elsewhere. We know no cause so sacred and no cause so urgent as that of safeguarding the workers from the calamity of being without hospital care when grievously ill. This many of them must be from January 16, 1913, when the National Insurance "medical benefits" will become due. We have appealed, and shall continue to appeal, with all the earnestness we possess to our colleagues throughout the press, quite apart from party—for this is not a party question—urging them to help us to bring to the knowledge of the people exactly the extent of the misery with which they are threatened. There seems no other course but to work together to secure the awakening of the people to plain facts, with the certain result that an arrangement must be come to between the voluntary hospitals and the Government before January 16 next. Such a step can secure to those insured persons who need it adequate hospital care without having to suffer the taint and degradation of charity which their German confrères by earnestness of purpose and independence have escaped.

In this connection we have to thank especially the editors of several of the great provincial daily papers, notably the *Birmingham Daily Post*, the *Leeds Mercury*, and the *Bristol Times*, to mention only three editors who are helping to save our workers from the cruelties which thousands of them must otherwise suffer. We know that politics and the war have made great claims upon the space of the newspaper press in this country during the last few weeks, but we ask every editor of every newspaper throughout the United Kingdom to bear in mind what infinitely vaster importance attaches to the solution of this question of adequate hospital care for the workers, for it must affect the homes of the working classes in the most tender and vital circumstances of family life and family security.

The great point which requires to be driven into the minds of politicians of all parties at the moment is that, they themselves are men in a highly responsible position. If they permit thousands of the working classes when in the direst necessity of illness to be without the adequate hospital care which they enjoyed before the passing of the National Insurance Act, they will be regarded deservedly by their fellow countrymen as unworthy of the trust which has been placed in them as members of Parliament. Mere justice must secure that he who fails to take action to prevent this threatened misery to possibly 30,000 insured persons each week will go down in history as representing a type of politician which no nation can suffer and prosper. We are especially indebted to the *Westminster Gazette*, which, of all metropolitan newspapers, has been the one to join hands with us in endeavouring to bring the facts to the knowledge of those whom they more immediately concern. We all have to face plain facts affecting the very lives of those who, without the help of the united press of this country, and of the responsible thinking men and women in it, may, as matters stand at present, be in a position almost if not quite as bad as that in which the unfortunate Turkish troops have been placed. It is unthinkable that England can exhibit to the world an even grosser neglect of prevision on the part of those responsible for the unavoidable deaths and miseries with which our working classes are at present threatened on and after January 16, 1913.

